# Low Cost Efficient Deliverying Video Surveillance Service to Moving Guard for Smart Home

**DOI:** 10.3390/s18030745

**Published:** 2018-03-01

**Authors:** Tatiana Gualotuña, Elsa Macías, Álvaro Suárez, Efraín R. Fonseca C., Andrés Rivadeneira

**Affiliations:** 1 Computer Science Department, Fuerzas Armadas University (ESPE), Av. General Rumiñahui S/N, 170501 Sangolquí, Ecuador; tmgualotunia@espe.edu.ec (T.G.); erfonseca@espe.edu.ec (E.R.F.C.); avrivadeneira1@espe.edu.ec (A.R.); 2 Arquitecture and Concurrency Group, University Institute of Cybernetic Sciences and Technologies, Las Palmas de Gran Canaria University, Lugar Diseminado Lomo Blanco, 11, 35018 Las Palmas de Gran Canaria, Spain; elsa.macias@ulpgc.es

**Keywords:** Smart Home, video surveillance, video streaming, WiFi, LTE, software design pattern, MVC, instant messaging, e-mail

## Abstract

Low-cost video surveillance systems are attractive for Smart Home applications (especially in emerging economies). Those systems use the flexibility of the Internet of Things to operate the video camera only when an intrusion is detected. We are the only ones that focus on the design of protocols based on intelligent agents to communicate the video of an intrusion in real time to the guards by wireless or mobile networks. The goal is to communicate, in real time, the video to the guards who can be moving towards the smart home. However, this communication suffers from sporadic disruptions that difficults the control and drastically reduces user satisfaction and operativity of the system. In a novel way, we have designed a generic software architecture based on design patterns that can be adapted to any hardware in a simple way. The implanted hardware is of very low economic cost; the software frameworks are free. In the experimental tests we have shown that it is possible to communicate to the moving guard, intrusion notifications (by e-mail and by instant messaging), and the first video frames in less than 20 s. In addition, we automatically recovered the frames of video lost in the disruptions in a transparent way to the user, we supported vertical handover processes and we could save energy of the smartphone's battery. However, the most important thing was that the high satisfaction of the people who have used the system.

## 1. Introduction

The Internet of Things (IoT) [1] has guided several smart projects in the last ten years: Smart Body, Smart Car, Smart Home, and Smart City…. The usage of low cost Wireless Sensors Networks (WSNs) together with embedded computers, has made possible the implementation of cheap video surveillance systems for the Smart Home. Smart sensors [2] can measure physical variables such as [3] temperature, humidity, pressure, direction and wind speed, lighting intensity, vibration, sound intensity, power line voltages, chemical concentrations, pollution levels, vital body functions, and gas concentrations…. Surveillance systems connect sensors to fiber optic, wired or wireless networks (Internet access networks) to evacuate data of interest to the guard. Those sensors can trigger the start-up of video surveillance cameras that register an intrusion in the Smart Home. The wireless communication between sensors (or video cameras) and moving guard is usually interrupted sporadically. We argue for intelligent communication protocols between sensors and guard to avoid problems of uncontrolled disruption of communication between them.

Video surveillance over the Internet is a real-time service that applies to very varied environments (underwater [4], Smart City [5,6,7], and Ambient Assisted Living for Smart Home [8,9,10] among others). We focus on Smart Home video surveillance assisted by sensors. We are not interested in monitoring people in the Smart Home as in the previous works. However, we focus in the detection of intrusion of people (thieves) in the Smart Home and rapidly communicate video information to the moving guards.

Video surveillance aims to ensure the security of goods and people in the Smart Home, as well as the tranquility of their owners. Video surveillance over the Internet has become one of the most demanded security services in last recent years. This has led to: (a) telecommunication operators proposing this service to their customers’ homes; (b) proliferation of low-cost video surveillance systems that any user can deploy; managing them through Web in a Smart Phone; (c) a mature industry appears producing technologies and proprietary equipment of very different type. Low-cost video surveillance systems (especially for emergent countries) can be deployed using embedded computers like Raspberry Pi [11] and open hardware like Arduino sensors. We take advantage of RaspBerry Pi and Arduino open hardware and free software to implement a physical demonstrator of our video surveillance system. When Arduino sensors detect the thief, a Pi Cam [12] will start recording the video in the RaspBerry Pi. Simultaneously the RaspBerry Pi will send an instant message (Telegram [13]) and an e-mail to subscribed guards (and the owner of the Smart Home). When any of those messages are received, the guard (or the owner) could start receiving the real time video. Depending on the location of the guard, it could use Long Term Evolution (LTE) or Wireless Fidelity (WiFi) to receive the video streaming.

Wireless Internet access suffers intermittent and sporadic service disruptions as is well known and we have shown it for LTE [14] and WiFi [15]. The causes of real time video streaming service disruption are widely known. Among those causes are the following:

- Coverage deficiencies in LTE (especially in emergent countries).
- Range coverage and radio channel interferences in WiFi [16], prolonged access to the channel (long periods of contention) of WiFi terminal.
- Bad planificafication of dense WiFi networks.
- Inefficient behavior of transport protocols of Internet in WiFi and LTE.
- Inefficient behavior of video servers (they usually do not detect efficiently if the wireless terminal effectively will receive video frames).
- Inefficient behavior of video clients that cannot process the deadlines associated to the reception and visualization of video frames.

The above adverse effects affect [17] the Quality of Service (QoS) enormously because video frames occupy the wireless channel but will be never received (they are lost) by the Smart Phone. This impedes other WiFi terminals’ access to the channel degrading the effective bandwidth. Moreover, the video server must restart the video session due to the fact it closes the open video session when the duration of the disruption exceeds a small (approximately 1 min) time limit. That restart of video session process in presence of several consecutive disruptions degrades enormously the Quality of Experience (QoE). This causes the guard rejects the service and the rapid battery consumption of the Smart Phone (receiving continuously repeated video frames). What is worse, the loss of very valuable images that can facilitate the early detection of the thief in the Smart Home or its surroundings.

We implemented our video surveillance system with open software platforms. Nevertheless, far from making a particularized implementation for our open hardware, we specified a software architecture based on software design patterns. Software design patterns are generic solutions for common design problems; they are reusable assets that help transfer knowledge from experts to newbies [18,19] and they are standard software design agreements. Our goal was to provide a general architecture design for the rapid development of open hardware and software video surveillance systems (assisted by sensors). In this way, we facilitate the implementation of systems based on different hardware and software platforms.

The main original contribution of this paper is the novel design of a video surveillance system for Smart Home that mitigates the adverse effects of disruption of the real time video streaming service. This system includes an intelligent protocol among sensors, video server and the video client used by the moving guard. The novel specification based on software design patterns allows any researcher to implement it for any hardware architecture using their own software components. We implemented our system in a very low cost hadware architecture using cheap sensors and camera. We built a Smart Home model for measuring QoS and QoE parameters, battery’s energy comsumption for WiFi and LTE, and the arrival time of notification of intrusion detection to the guard wireless terminal. The experimental results showed the small time needed for the guard could come to the rescue while he can view video of the intruder’s actions. This is a novelty focusing in the surveillance system based on sensors, which have had a high interest of system testers (QoE).

In Section 2, we review the background of the real time video streaming surveillance and related works. In Section 3, we state the main ideas of the system architecture and its operation. We present the software pattern design, and the open hardware and software implementation of the prototype. In Section 4, we present an indicative formal model that informs the reader about the adverse effects of video service disruptions and how they affect the QoS and the QoE. In Section 5, we present the discussion of the experimental results we obtain after many tests of our prototype implementation. Finally, we sum up some conslusions and future work.

## 2. Background and Related Work

Firstly, we review some works on video surveillance service to state the main idea and aspects related to our system. Secondly, we review works on mitigating the adverse effects of service disruptions and differences with our method and finally, we review methods for programming systems similar to ours. The current video surveillance systems are very sophisticated and propose a video treatment focused, among others, on:

- Intelligent image processing. In [20,21] video surveillance systems are focused mainly on the detection of activities such as fights, assaults or excesses that may occur within an environment. The system contains external sensors that allow capturing information from the environment using cameras, microphones and motion detectors. When a security alert occurs, a centralized broker, for example will call the police, or blocks doors.... It does not support smart phones so it does not mitigate adverse effects of service interruptions. In [22] authors proposed a prototype whose intrusion alerts allows the detection of movement and location of objects in a determined area by processing the video coming from different cameras. Our system detects movement of the thief but we are not interested in his exact localization. In [23] a system capable of processing images was presented to obtain relative positions of objects supporting environmental adversities and combining it with IoT to improve the acquisition of information. Kavi and Kulathumani [24] are able to detect orientation of objects. We are not interested in the segmentation of objects or the affectation of environmental conditions, but in a real-time reception of images that facilitates a first vision on the part of the guard who observes the intrusion in the Smart Home. In [25] is treated the problem of intelligent recognition of objects in nigthtime using visible light cameras. They proposed an interesting image recognition system for near infrared cameras that can operate in daytime and nighttime. Whichever type of camera is used the problem of video service disruptions still exists. That is the reason why we only considered visible light cameras and full delivery of video. In [26] authors proposed a solution through the design and development of a video surveillance system, which uses semantic reasoning and ontologies. This system is able to work with small and cheap cameras, reduce required bandwidth and optimize the processing power. Our system also is able to use ontologies over an embedded processor.
- Smart codification and compression of objects. The main idea is to reduce the needed communication bandwidth between server (that processes a high amount of videos) and client, sending only relevant information to the client (user) [27]. Due to the low economic cost we imposed on our system, we consider a small number of video cameras, so we did not need to implement a system of this type. In [28] was presented a system to extract metadata of important objects to avoid the impossible to solve problem of monitoring large number of cameras. We did not treat with this problem due to the reduced number of cameras we considered.
- High number of video streams synchronization. Pereira et al. [29] proposed a window strategy together with a correntropy function to synchronize video streams of line applications that require a low computational power. We did not focus in synchronization of video streams; on the contrary, we focused on the adequated reception of frames of a video stream. However, the application of that technique to the synchronization of several video streams in our system requires a deep study to solve the mitigation of adverse effects of several video streams at the same time.
- Usage of low economic cost and embedded computers for hosting the video streaming server, which connects to a video camera through Universal Serial Bus (USB), and uses a mobile network for communicating the video streaming [30]. In [31] is used a built-in system based on the ARM9 processor (freely available), a 3G mobile network card, a USB camera that captures video using the H.264 standard and sends it to a video server. The user accesses it with an *Android* smart phone. The construction of an embedded system as a server and video processor is cheap because there are low economic cost freely distributed hardware and software to do it. However, in [31] they do not focus, as we did in real time streaming video. We used a general-purpose embedded computer (Raspberry Pi) for the good performance it offers and its low economic cost.

Current research in video surveillance systems does not focus on the communication of real time video to the guard’s wireless terminal (while he moves). This is due to the fact that usually, that video is received on a desktop computer (or specialized fixed terminal) and the physical level of the communication network is mainly optical fiber-based. We focused into the exploration of the communication of the video using a wireless access network to Internet kernel. That is, both the video server and the guard’s wireless terminal connected to a wireless network (LTE or WiFi). The mobility of the guard is essential for early acting in the Smart Home just in case he is close to the Smart Home. If the guard could use a WiFi network (for example, he is within an urbanization area), he would experiment video service disruptions. On the other hand, if the guard is not close to the Smart Home, and he wants to move rapidly to the Smart Home, while a colleague of him is visualizing the video, he would use LTE (also probably experimenting video service disruptions), to early visualize the movements of the thief. For both cases, the aim of our video surveillance system is to mitigate the adverse effects of real time video streaming service disruptions. In the same way, the smart phone of the guard must need to do vertical handover [32] between LTE and WiFi (or viceversa). In this case, the wireless network must implement rapidly the handover processes. We must assure that the smart phone is able to implement rapidly those network functions.

To mitigate the adverse effects of video disruptions, there are several techniques, which we present next. We distinguish adaptive video streaming algorithms into three categories:

- Based on the regulation of the transmission rate. They estimate the highest possible rate of video transmission [33].
- Based on adaptive video buffer. They seek the relationship between the occupation of the buffer and the selected video bit rate and the available bandwidth [34]. Latency would increase for real-time video. Our system must minimize the latency so this scheme would be limited. In [35] the starting latency, the reproduction fluidity, the average reproduction quality, the smoothness of the reproduction and the cost of real time video are improved. In [36] was managed the bandwidth tolerance of the QoS degradation.
- Based on prediction of QoS parameters, which optimizes the allocation of resources and related variables in a control model such as the Markov Chains [37].

Most of the above algorithms keep multiple copies of video content with different bit rates, which create a huge storage load on the video server. In addition, video transcoding is expensive and difficult to apply for real time video streaming. These are two important problems for our system of low economic cost and calculation power, so it was not possible to use them. However, the main problem we wanted to solve was the fact that the server closes the video session and the client must start again to download the video from the beginning. When this problem occurs, the previous techniques do not solve it.

We next present other particular solutions.

The authors of [38] proposed a specific solution for real time video streaming in a mobile teaching domain. Intelligent software agents detected the video streaming service disruption. When the server detects the disruption it stores the video it was sending to the student, which can retrieve the class from the disruption point. This solution was designed for a particular firm real time service (video surveillance is a soft or hard real time service).

Several authors [39,40,41] have presented mechanisms to reduce the effect of video service disruptions in wireless environments and to maintain the continuity of the on demand streaming video service, for mobile phone. However, those proposals do not apply to real time streaming video. 

We have used the Proxy software pattern to mitigate disruptions applying that technique to different smart phones and operating systems [42,43,44], supporting real time communications among Web browsers and for the videoconferencing service [45]. We used Web Real Time Communication (WebRTC) framework [46], to implement the Web communication system recovering disrupted sessions of real time video streaming service between Web browsers. Our technique Automatic Reconnection WebRTC (ARW) used tools of WebRTC to obtain traces of QoS from the WiFi channel to manage disruptions [47].

In this paper we refine our previous unpublished studies in [48,49] improving the high level abstraction model of performance, the software pattern design and showing new results, which demonstrate the effectiveness of the mitigation technique for early arrival of video to the guard, arrival of notification messages of intrusion, handover support and battery energy consumption.

Next, we review several similar works and analyze the contribution of the paper regarding the specification of the software architecture based on software design patterns.

Sanchez et al. [50] presented a hardware and software architecture for a video surveillance system. They did not specify the software architecture at design level. This is an example of a very particular software architecture implementation. The result is that it is very difficult to reproduce the software in other hardware platform. Our intention was to make a general specification implementable in any hardware platform. In this line, we followed the idea presented in [51] that made an interesting discussion about the utilization of software architectures for Health Care in the Smart Home and presented an application example. We have done a pioneer adaptation of that idea for video surveillance systems in the Smart Home.

The design of software based on software design patterns improves the quality of the artifacts produced and reduces the costs of software maintenance [52,53]. In addition, the analysis of the interrelationships of software design patterns in an application would make a more concise and more cohesive design than the application of patterns in isolation [54,55,56]. Several works study the impact of software design patterns on software quality (maintainability, reliability and proneness to errors [57]) implementing several experiments. The authors of [58,59,60] researched the energy consumption of the software design patterns. In [48] we did an exhaustive analysis for the formal derivation of the best software design patterns for a video surveillance system based on alarms of sensors in a Smart Home. In this paper, we seek to expand that derivation taking into account the smart phone energy consumption. We have treated to follow the guide provided in [61,62].

## 3. System Architecture and Operation

**Figure 1:**
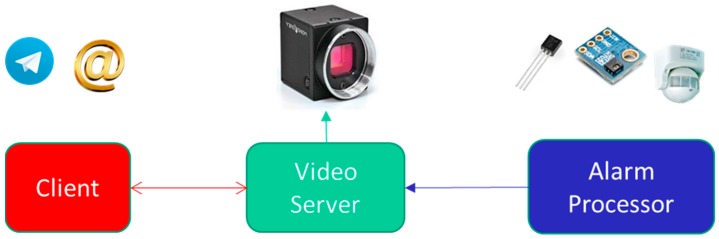
Scheme of main components of the video surveillance system.

Figure 1 shows a schematic of the main components (black box model) of our video surveillance system: sensors, sensor alarm processor, real time video streaming server and client.

Broadly speaking, the video surveillance system has the following main operations:

- The sensors are continuously sending sensed data (with a certain sampling period) to the alarm processor.
- The video camera starts operating once the Alarm processor fuses the sensor data and determines that an intrrusion has occurred. At that moment, the video will be stored in the video server memory (a file that works as a buffer) and simultaneously an instant message (Telegram application) and/or an e-mail are sent to the Client (guard). The file containing the recorded video can be used before the judge.
- The Client will receive a Telegram instant message and/or an e-mail. Whenever he wants, he can start a video streaming session on the Video Server, clicking a link (containing the *Internet Protocol Address* (*@IP*) of that server) in the instant message or in the e-mail text. If the Client receives the video while the server is producing it, the video will be delivered in real time. However, the Client could start an on demand video Streaming session whenever he wants, once the Video Server finished recording that video.
- Video service disruption: The Video Server registers the last set of frames sent to the Client continuously and has not been consumed by the Client. When the Client experiments a service disruption, the Video Server will continue sending from the last set of video frames previously sent. There is no need to restart the video session because the Video Server also maintains the original session opened during at most 10 min (the time that lasts ussually an intrusion). In that simple way, nested disruptions can be easily managed.

**Figure 2:**
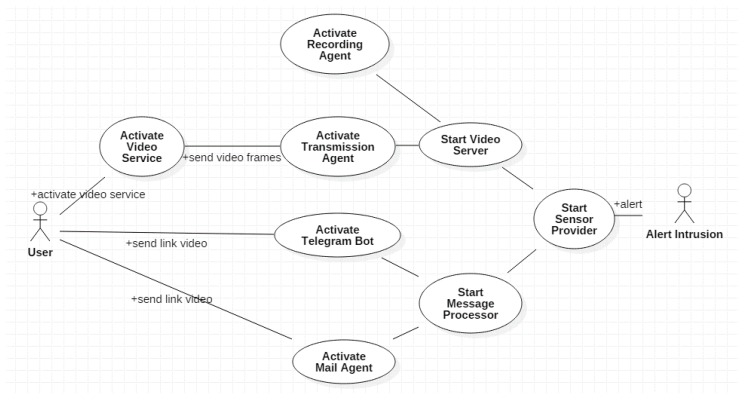
Diagram of actors and use cases of the video surveillance system.

An actor (represented as a small doll) is a person or other entity that interacts with the system been described. A use case (represented as an ellipse) describes the way an actor interacts with the system to reach its objective. Table 1 presents the behavior of actors and use cases. Figure 2 shows the diagram of two actors and eigth use cases for our system. Start Sensor Provider tests the sensors notifying, in parallel, the Start Messages Processor and Start Video Server use cases when an intrusion will be detected. Then (a) the Start Video Server triggers the recording of the video of the intrusion (Activate Recording Agent); (b) Start Messages Processor triggers the Activate Telegram Boot Agent and the Activate Mail Agent, which provide Uniform Relocator Locator (URL) links of the video to the User. Once the User clicks on those links (Start Video Service), he will receive the video streaming (Activate Transmission Agent).

**Figure 3:**
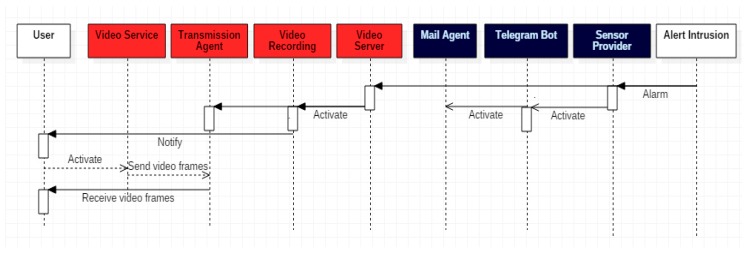
Sequence diagram of the real-time communication protocol of the intrusion video once the sensors have been triggered.

Figure 3 shows a simple sequence diagram in which repeatedly, the Sensor provider receives and processes all the alarms generated by the sensors (different intrusions), the Message processor is notified to construct the message and the agents Telegram and Mail send the messages to the Client. The sensors work independently to the Video Server. That is, detected an intrusion, the alarm would occur and until the intruder’s movement ceases, no new alarms will be generated (it is not considered the unlikely event that there is an intrusion and a new one occurs before the first one ends).

**Figure 4:**
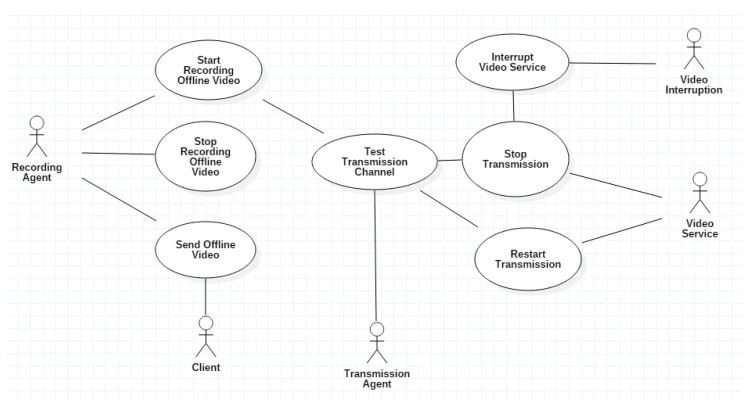
Diagram of actors and use cases of the video surveillance system when an interruption occurs.

Figure 4 refines the occurrence of a real time video streaming service disruption with four use actors and seven use cases. Table 2 presents the behaviour of actors and use cases for Figure 4. Transmission Agent continuously is testing the wireless channel (Test Transmission Channel). In parallel, the Video Interrupt actor controls a video service interruption. When a video service interruption is detected, the Video Service will stop (Stop Transmission) and in parallel Recording Agent will start recording the video (Start Recording Offline Video). When the interruption finalizes, the transmission is restarted (Restart Transmission) and in parallel the video offline recording stops (Stop Offline Video Recording). The Client can demand the offline video reception once recovered the video service interruption (Send Offline Video).

**Figure 5:**
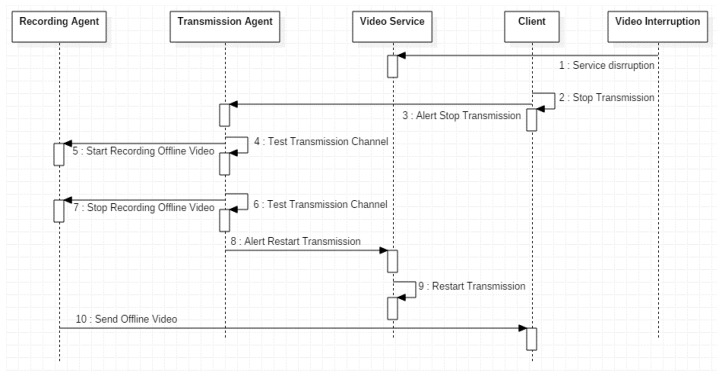
Sequence diagram for the treatment of a real time video streaming service disruption.

Figure 5 shows the sequence diagram of the treatment of a real time video streaming service disruption. The Video interrumption communicates to the Video Service the disruption, which will make a forward to the Transmission Agent so that the Recording Agent begins the recording of the video. The Transmission Agent continues checking the status of the wireless channel until the communication can be re-established at which time the video is still sent, in real time, to the Client.

In [48] we presented all the sequence diagrams among the objects that implement the components of the system according to the software design patterns used. We present the formal derivation of software design patterns in the next section.

### 3.1. Software Design Pattern Specification

The appropriate selection of patterns must take into account the cases of use and quality requirements of the software, and the technology chosen [18]. The software design pattern named Model-View-Controller (MVC) allows separating the application data, the Client interface and the logic (control). That allows making changes and adding new functionalities easily in different incremental prototypes. In addition, it supports the integration and reuse of components, provides scalability and eases the prototype development. The most abstract vision of any video surveillance system would start from the MVC:

- Model (Server): it is located in the Video Server (video buffer that stored temporally the pending video still not sent to the Client and other stored videos). The Model consists in an Observer software pattern in charge to control de videos. An external Observer software design pattern abstracts the data model corresponding to the sensed data. That Observer is constantly testing the sensor data communicated to the Controller.
- View (Client or user): It consists of several views: the video display, the e-mail and Telegram messages interfaces and the links that allow starting the video streaming. Other simple view is the warning of disruptions.
- Controller: It is the most important software pattern. It is in charge to do all the control of video service disruptions, the control of the wireless channel to advertise possible disruptions and the cooperation with the sensor alarms. It consists in the cooperation among different standard software patterns that we present next. We distinguished internal software patterns to the Controller (inside the MVC) and other external software patterns to the Controller (outside the main MVC pattern).

**Figure 6:**
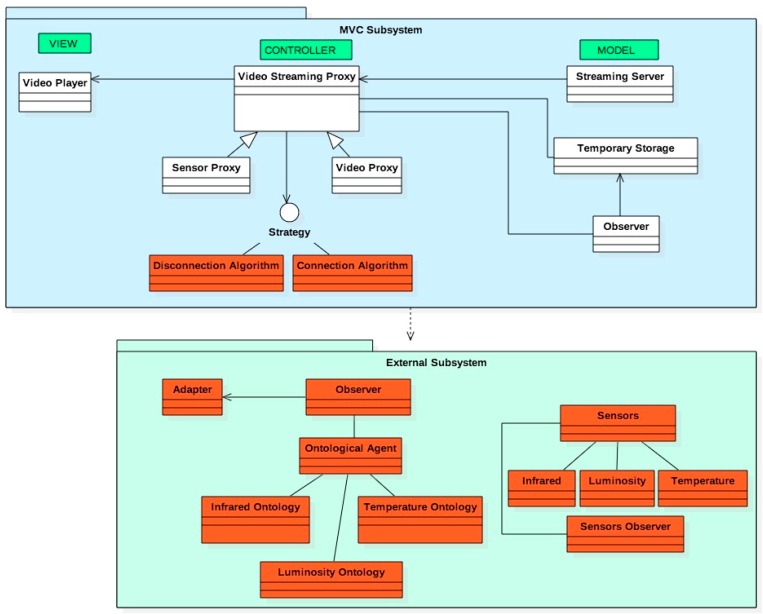
General software design pattern of the video surveillance system.

The internal software patterns are Proxy, Strategy and Adapter. The first implements the actions of our previous solutions (coordinate the Model and the different views). In addition, it is in charge to test the state of sensors, to fuse the sensed data and to determine if an intrusion has occurred (received from external Observer). If a video service disruption occurs, it will invoke the Strategy, which will implement a particular algorithm for treating the disruption. This is very flexible design of software because the treatment of the disruption can be changed without changing the overall video surveillance system. The Adapter (ontologic software design pattern) receives data from the external Observer. That is, the sensed data are communicated to the Adapter which, can convert those data into the corresponding data service (we implement Web Services). The flexibility of this design decision allows the designer to change the communication of sensed data without change the overall video surveillance system. Figure 6 shows the general design based on software patterns and the relation among them. 

### 3.2. Low Cost Hardware Implementation

**Figure 7:**
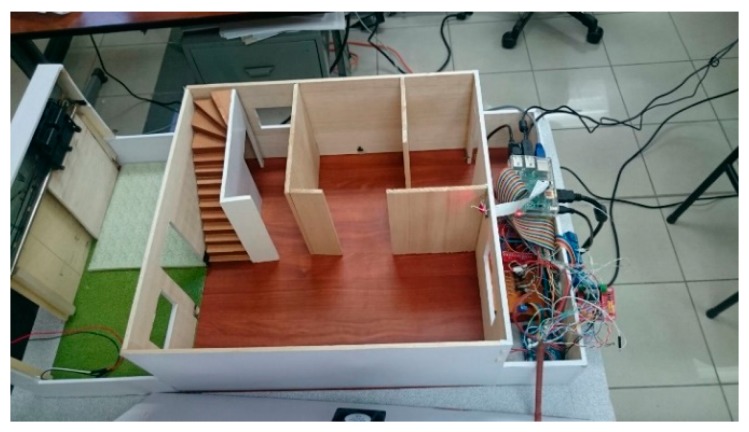
Photograph of the model built to emulate unwanted intrusions in a Smart Home.

We built a physical model that emulates a typical Smart Home with several rooms, garage, stairs… (Figure 7). Within that model, we installed the sensors and the video camera to generate intrusion alarms and to record the corresponding actions. Inside 2 light sensors were installed in the front and back of the Smart Home, 2 temperature sensors in the living room and kitchen, 4 infrared sensors for motion detection (front, rear, kitchen, bathroom) and 2 actuators for cooling (fans). The technical characteristics of the sensors used are:

- Ligth: Dimensions 65 × 11 × 13 mm, series Photoresistor-BH1750, measurements 1-65535 LX, sampling 2 s.
- Digital temperature (Ds28b20): Each device has a unique 64-bit serial code stored in its Read Only Memory (ROM), multipoint capability that simplifies the design of temperature detection applications, can be powered from the data line. The power supply range is 3.0 V to 5.5 V. Measures temperatures from −55 °C to +125 °C (−67 °F to +257 °F) ± 0.5 °C to the nearest −10 °C at +85 °C. The resolution of the thermometer is selectable by the user from 9 to 12 b and converts the temperature into 12 b codes in 750 ms (maximum).
- Infrared Barrier: 10.2 × 5.8 × 7 mm phototransistor, peak operating distance: 2.5 mm, operating range for collector current variation greater than 20%: 0.2 mm to 15 mm, typical output current under test: 1 mA, ambient light blocking filter, emitter wavelength: 950 nm.

An Atmega 328 arduino microcontroller did the processing of the sensed data. We used a 20.7 Mpixels camera, LED flash, ½ 3 ′ ′ sensor, geotagging, and image stabilizer. To implement the videostreaming server and other storage control software, a RaspBerry Pi B+ was used with a 900 MHz quad-core ARM Cortex-A7 processor, 500 MB of RAM, 4 USB ports, 40 GPIO pins, interface of the camera (CSI), screen interface (DSI), Micro SD card slot and VideoCore IV 3D graphics core.

### 3.3. Software Implementation Based on Agents, Web Services and Free Platforms

**Figure 8:**
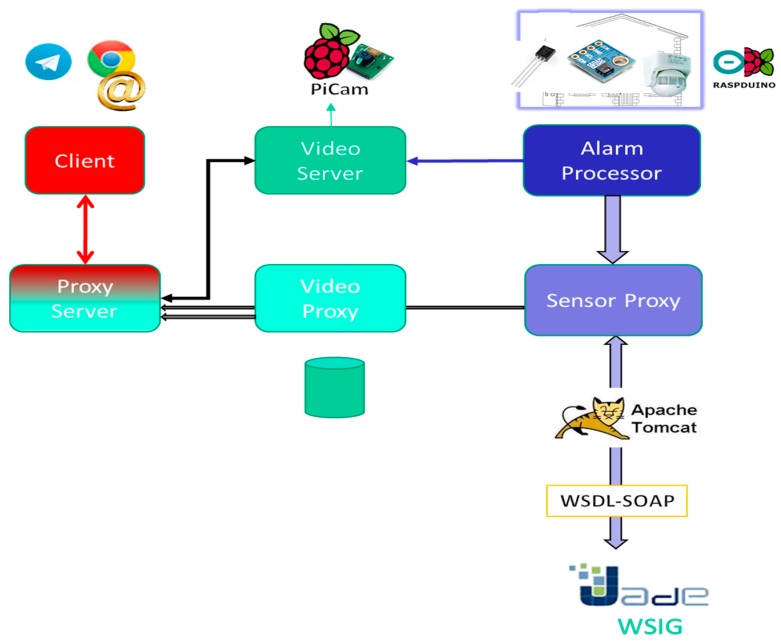
Scheme of the use of free software for the implementation of the components of the video surveillance system.

We made use of Service Oriented Architecture (SOA) for implementing the software specified by software design patterns. Avila et al. [63] introduced SOA and reviewed its applicability in the field of IoT for telemedicine and Healthcare. We did not know other researchers that have applied that concept to video surveillance systems similar to ours. We used the Multi Agent System (MAS) platform named Java Agent DEvelopment Framework (JADE) and Web Service Integration Gateway (WSIG) [64] for implementing the video streaming service disruption proxies. This decisition is in the line of recent approaches to software design for IoT in Smart Home control [65,66]. We do not know any other work that has used MAS for video surveillance systems programming able to interoperate with Web services in the domain of Smart Home. Figure 8 shows a diagram of the different free software components used for the implementation of the video surveillance system.

With Raspduino, the Arduino sensor platform was interoperated with the RaspBerry Pi B+, enabling data to be collected: The sensor's identifier, the sensing measurement and a time stamp for that sensed value. These values are sent to a proxy capable of interacting with JADE agents. We defined the agents’ behavior which infere ab-normal situation (intrusion) based on the sensors’ values. Since JADE originally used its own ontology for the message passing, it was decided to make this communication interoperable through Web services. That is the reason why we installed WISG addon, which transforms those messages into Web services. With Web Services Description Language (WSDL) and Simple Object Access Protocol (SOAP), the data were passed to the Tomcat Web Application Server. Tomcat is necessary to support a Web browser compatible with the reception of video in Hiper Text Markup Language (HMTL) 5.

If an alarm was generated, the PiCam would be triggered to record what happens in the Smart Home. That camera connected to the RaspBerry Pi B+ video streaming server called RPiCam, which did not record audio natively, but it recorded video in the Motion Joint Photographic Expert Group (MJPEG) version 4. It communicated video in H.264 format that can be received in HTML5 in the Web browser (which can be anyone who accepts video in HTML5: practically any current browser). 

## 4. High Level Abstraction Model of Performance

We focus now in the video service disruption performance. We first model the occurrence of disruptions not mitigated. The second model corresponds with the mitigation of service disruptions with our technique. We compare the results of both models with the case in which no video service disruptions occur. The objective is to show informally the impact of our technique in QoS and QoE.

### 4.1. Formal Model for Video Streaming without Service Disruptions Mitigation

Let us analyze the impact on QoS and QoE with a simple model abstracting details of the communication and observing only the amount of time and energy needed to send all the video. Figure 9 shows a diagram that facilitates the understanding of the model. If there is no video service disruption, the Client (*C*) will take *T_s_* time units (t.u.) to start the video streaming session and it will indicate the beginning of the reception of video packets (*SETUP*, *OK* and *PLAY* are the tipycal commands in *Real Time Streaming Protocol* (*RTSP*)). After *t_p_* t.u., *C* will receive the first video package. From there, *C* will receive a new package after each *d_i_* t.u. (delay of arrival of package *i* with respect to package *i* − 1, where *i* − 1 and *i* are independent on the numbering of packets in the server). Therefore, the optimal time (*T_opt_*) that *C* takes to receive the whole video is the one shown in Equation (1):

(1) $$T_{opt}=T_{s}+t_{p}+\overset{n}{\underset{i=2}{\sum }}d_{i}$$

where *n* is the total number of sent packets, *T_s_* is the initialization time of the session and *t_p_* is the arrival time of the first packet. 

In case there are *m* video service disruptions (*Iz, z = 1..m*) then the time (*T_int_*) it takes to receive the video will be considerably greater and is a function of the number of disruptions that occur and their duration (*Ti_j_*), as indicated in Equation (2):

(2) $$T_{int}=\left(T_{s}+t_{p}\right)m+\overset{m}{\underset{j=1}{\sum }}Ti_{j}+\overset{K_{i}}{\underset{i=2}{\sum }}d_{i}+T_{opt}$$

where the factors *K_i_* are the numbers of packets received before the video service disruption *i* occurs and we assume that at least one is received before any video service disruption.

An important adverse effect is the number of times a video packet is retransmitted. Equation (3) gives it:

(3) $$\overset{K_{i}}{\underset{i=2}{\sum }}d_{i}$$

In each of these retransmissions the packet is occupying the WiFi channel, preventing other packets from being communicated (thereby increasing the contention in the channel). In addition, if we assume that the smart phone has an expenditure of *p* units of energy consumption (u.e.), then the smart phone will be making an expense (extra inefficient battery consumption) indicated in Equation (4):

(4) $$\overset{K_{i}}{\underset{j=2}{\sum }}p$$

The most important adverse effect is the degradation of QoE [67]. It is possible that the user accepts that the same packets were retransmitted once; but he would not be willing to have it retransmitted more than three times. In addition, most likely he could decide not to continue restarting video streaming sessions. This is important because in a video surveillance system like the one we designed, it is precisely a distinctive element that the user can make a visualization of the video as soon as possible while, for example, he is on his way home.

This is not a particular problem of our system, but occurs in other scenarios. For example, in [68] authors mathematically demonstrated that at the link level, disruptions whose duration varies between many seconds or a few minutes produce buffer underrun and it is proposed to mitigate them by buffering control, monitoring the network and regulating the injection of video packages from the Server. At the link level, this control can work, but when the disruptions have a certain duration, the problem is that the video streaming servers abort the video session and must be restarted as we indicated above. In [69] authors analyzes the optimization of the use of video caches in order to minimize the video disruptions and more specifically to minimize the *T_s_*. In [70] a study of the implementation of a video surveillance system on LTE was presented in which it is recognized as one of the lessons learned that an appropriate buffering system must be provided in order to support real time video streaming without disruptions, in addition to modifying the Android buffering system. We in [48,49] analyzed the use of several video streaming buffers and the use of video streaming server proxies that allow monitorizing communication parameters between the client and the server to minimize the *T_s_* and the retransmissions of packets. In addition, we automated the reconnection of the video streaming session to avoid the user having to do it manually. Thus, we reduced the adverse effect of video service disruption in the QoE. Our schemes can be adapted to build the solution presented in [70] taking into account the specific mechanisms of QoS.

### 4.2. Formal Model for Video Streaming Mitigating Service Disruptions

Figure 10 shows a time diagram invested in the video streaming system with our mitigation mechanism. All video packets are stored in the buffer the first time they are sent. When the disruption *I_1_* occurs, the packets are still stored until all the video packets are completed. To prevent the *C* from sending an order to stop the video streaming, we choose to store the video for a predetermined but configurable amount of time (between 8 and 10 min, because this is the estimated length of intrusions in the Smart Home). At that time, the *n* packets of the video collected by the camera would be stored once the alarm server produces a new alarm.

As verified experimentally by Santos et al. [71] *T_s_* is one of the most important performance factors in video streaming. They compare the efficiency of RTSP and WebRTC for video streaming concluding that the second is more inneficient than the first. Thus we are in the correct line trying to avoid the *C* must restart the video session each time a video service disruption occurs.

With the new mitigation technique, the time to send the full video is given by Equation (5):

(5) $$T_{buf}=T_{s}+t_{p}m+\overset{m}{\underset{j=1}{\sum }}Ti_{j}+\overset{n-m}{\underset{i=2}{\sum }}d_{i}$$

With which, from Equations (1), (2) and (5), Equation (6) relates the different delivering times:

(6) $$T_{opt}<T_{buf}\ll T_{\mathrm{int}}$$

which indicates that the user would save a considerable amount of time in watching the video in the presence of disruptions in relation to if our method will not be applied. In addition, it provides greater comfort because the system makes reconnection automatically. This undoubtedly improves the QoE.

Another important aspect is the battery energy consumption. With our mitigation mechanism, it is possible to minimize the extra energy consumption in retransmissions. Indirectly, this also improves the QoE because the user has more time to connect the video surveillance system. On the other hand, it is important to maximize the life of the battery to ensure greater tranquility of the user in such a way that it does not perceive it can disconnect from the system and not know what is happening in the Smart Home.

## 5. Experimental Results: Discussion

To verify the operation of the video surveillance system, we implemented multiple tests. With those tests, we tried to observe the response times for the mitigation of disruptions of the real time video streaming service; the time of arrival to the client of the notifications via e-mail and Telegram; and the extra battery consumption due to real time video streaming service disruptions.

### 5.1. Discussion about QoE Performance

It is widely known that the optimization of the measurement of the QoS parameters is not enough to ensure a general satisfaction of the user, because each person is different and has different expectations of quality. For this reason, the International Telecommunication Union (ITU) has published a new set of ITU-T recommendations focused on parametric QoE monitoring of video services [72,73]. We focus on Mean Opinion Score (MOS) to observe the QoE for being a subjective metrics that complements the interpretation of QoS parameters.

According to [74] the sample size must include 5% of error, 95% of confidence and 50% of heterogeneity. Taking those aspects in mind, we considered 25 people (16 men and 9 women to reflect the fact that usually there are more men than women dedicated in guard profession). We were interested in the usability of the mobile application and video service interruptions. For that reason, we choose young people between 18 and 24 years old, undergraduate and normally user of Internet video streaming services. This sample can be considered the most critical one for evaluating video service interruptions because they have a lot of experience with video service interruptions and mobile applications. Their general cultural background is considered homogeneous because all of them live in Quito (Ecuador). However, their specific technical profile varies because they study different university grades (Computer Science, Law, Biology…). 

We used the ITU P.800.1 for the opinion scale, and we asked the following questions (using a Google Forms form):

Q1. How would you rate the design of the user interface of the application?
Q2. How would you rate the usability of the application?
Q3. How would you rate the interactivity of the application?
Q4. Did you have problems accessing the application?
Q5. Did the application have an execution error?
Q6. Did the application reconnect the video without reloading the page?
Q7. Did the application send e-mail and Telegram notification messages?

The response classes for questions Q1, Q2 and Q3 were Excellent, Very good, Good, Improvable, Very improvable. The response classes for the remainder questions were Yes or No. Respondents answering yes to Q4 and Q5, were asked to identify the problem. We did that to have feedback and improve the application and on the other hand, to ensure that the responder answered conscientiously. Only two people contributed feedback.

**Figure 11:**
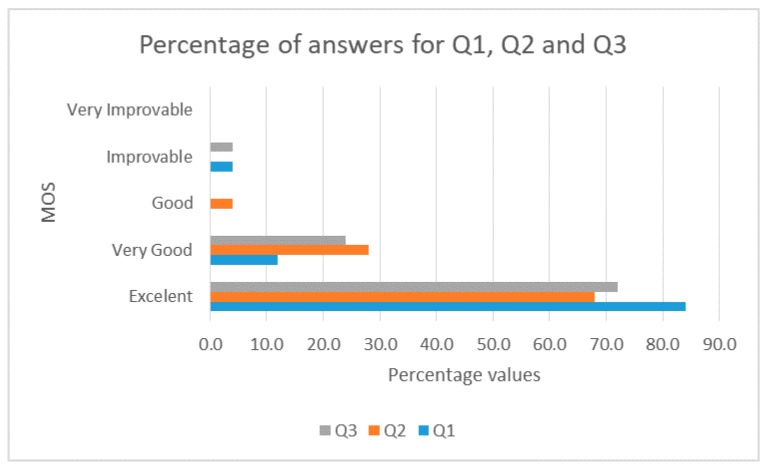
Percentages by categories of the MOS for Q1, Q2 and Q3.

Figure 11 shows the percentages of responses to Q1, Q2 and Q3 in each category of the MOS. The QoE is Excellent or Very good in most cases and there are not Very Improvable cases. Another important factor is the homogeneity of the values for the three questions. Therefore, we can assure that the design of the interface, the usability and the interactivity of the application of the video surveillance system is Very good or Excellent.

**Figure 12:**
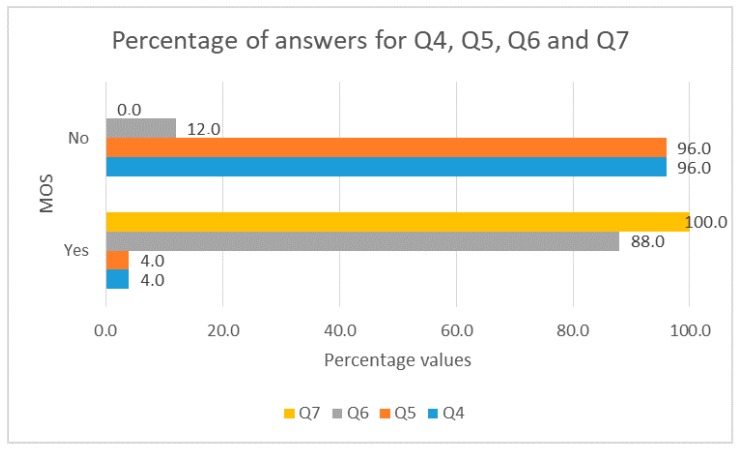
Percentages by categories of the MOS for Q4, Q5, Q6 and Q7.

Figure 12 shows the percentages of responses to Q4, Q5, Q6 and Q7 in each category of the MOS. The QoE is very high in most cases. All notification messages (e-mail and Telegram) reached users, so there is no risk that a guard is uninformed of a possible intrusion in the Smart Home. The errors and problems of access to the video surveillance system are very few, so the video surveillance system is accessible and works excellently. The answers to Q6 are the most important because they focus on verifying if, specifically the mitigation of adverse effects of the real time video streaming service disruptions will correctly be done (making the video recover from the point where it was disrupted). The operation is very good. This is important because in most cases the guard would not have problems accessing the video of the intrusion.

### 5.2. Discussion about QoS Performance

As mentioned in Section 4.1, there are key parameters (according to [75]) to define the efficiency of the QoS supported by our video surveillance system (assisted by sensors): *T_s_* (initialization time of the session) and *t_p_* (arrival time of a packet). We call user Waiting Time (uWT) the delay time in starting the video playback in the Client since he requested to open the video session (issue of a *SETUP* command in RTSP). That is, *uWT* is the time the Client (user, guard or owner of Smart Home) waits to receive and visualize the first set of video frames. Abstracting insignificant values of computation in the wireless terminal, we could consider:

(7) $$uWT=T_{s}+t_{p}$$

The *uWT* < 10 s (according to [75]) and because nobody, among the interviewees (informally questioned), was willing to support greater values.

The percentage of video packets loss (*vpl*) could explain part of the disruptions (*Iz*) and its duration, the jitter (*d_i_*) and factor *K_i_*. We are not interested in verifying our high level abstraction model, but we consider very important to obtain the values for *vpl* in order to show if it will be less than 25% (*vpl < 25*), according to [75].

The playback of video was embedded in a Web page (Web View). The Web page must be reloaded just in case the Video session must be restarted (long disruption or any other bad event occurred). We name this parameter *WpR* and we considered that if it is greater than 3 (*WpR > 3*), then adverse effects will occur and the mitigation mechanism has not worked well. We obtained this limit from the experience with interviewees (informally) in QoE measurements.

We tested those parameters making a full campaign of experiments considering several disruption patterns. We did six blocks (*blk_b_*, *b = 1..6*) of five tests (*tst*) each. In each block, we considered different amount of disruptions. Specifically, we considered one disruption in *blk_1_* and *blk_4_*, two disruptions in *blk_2_* and *blk_5_*, and three disruptions in *blk_3_* and *blk_6_*. In this way, we wanted to do a full test of the mitigation of adverse effects of our video surveillance system. We used a 300 s video and intentionally provoked the disruptions moving along a full range and shadowed WiFi zones. 

We also considered different kinds of wireless terminals to observe the influence of video consumption and the wireless communications:

- WTerm 1: Smart phone Celular Sony Xperia Z3 Compact; 127 × 64.9 × 8.6 mm; 1280 × 720 pixels (4.6′′); Android 5.0; processor of 4 cores (2.5 GHz), Graphic Processor Unit Adreno; Battery capacity 2.600 mAh; 2 GB RAM; LTE and WiFi (IEEE 802.11 g/n/ac). This terminal was used in the *blk_i_* (*i* = 1..3).
- WTerm 2: Laptop Mac Book Pro 15′′; 1920 × 1200 pixels (15.4′′); Mac OS High Sierra; 2.2 GHz quad-core Intel Core i7, Turbo Boost up to 3.4 GHz, with 6 MB shared L3 cache; 4 GB de SDRAM DDR3 (two moduls SO-DIMM de 2 GB) to 1.333 MHz; WiFi (IEEE 802.11 g/n/ac). This terminal was used in the *blk_i_* (*i* = 4..6).

**Figure 13:**
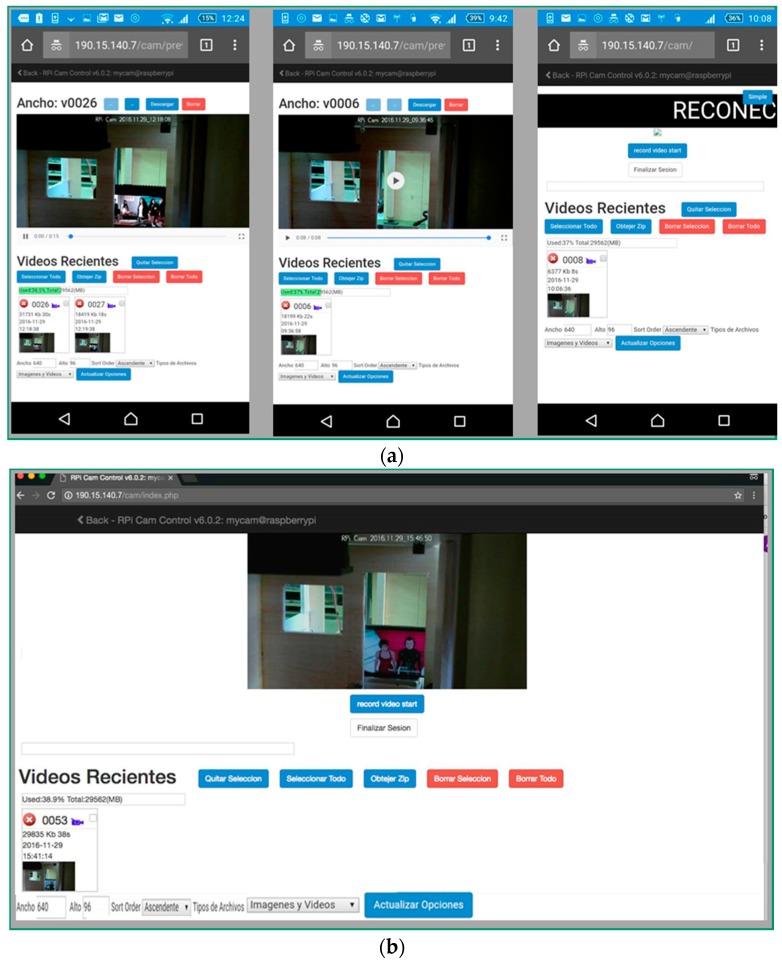
Client interface (in Spanish) of the video surveillance system for the WTerm1 and WTerm 2: (**a**) Three different views for Wterm 1; (**b**) One view for Wterm 2.

Figure 13 shows the Client interface (in Spanish) name Recent Videos (Videos Recientes) of the video surveillance system for the WTerm 1 and WTerm 2 (the user has previously clicked in the link it received in the e-mail or Telegram notification message). That interface consists on a list of videos of recent instrusion in the Smart Home (each video is an un-disrupted part of the video of the instrusion). The user can decide to watch each video (first two window captures in Figure 13a) or automatic reconnection (last window of Figure 13a). That interface consists in a responsive Web application: The Web page automatically renders elements in it depending on the kind of terminal. This view has typical option to manage the stored videos in the video server (located at @IP: 190.15.140.7): select all (seleccionar todo), remove file (borrar selección), remove all (borrar todos), end session (finalizar sesión)… with those options the user can manage his subscribed videos for a particular Smart Home. The option *< Back − Rpi Cam control* allows the user to return to message notifications view.

We used *Google Chrome* browser (version 54.0.2840.85 for WTerm 1 and version 54.0.2840.98 for WTerm 2), and tcpdump version 4.3.0, and Wireshark version 1.11.1 for capturing traffic in the video server.

The place in which we did the test was the campus of the Escuela Politécnica del Ejercito (ESPE) in Sangolquí − Ecuador, closed to the installations of Computer Science Departament (longitude: −0.312981, latitude: −78.445633 in Google maps) in the uppest floor of the building. Several buildings and shadowed zones for WiFi and LTE surround the Department building. It is very appropriated to show the utility of our video surveillance system because a notification message can arrive to the Smart Phone of the guard that is in the campus and he will cross several WiFi shadowed zones before reaching the Smart Home model.

**Figure 14:**
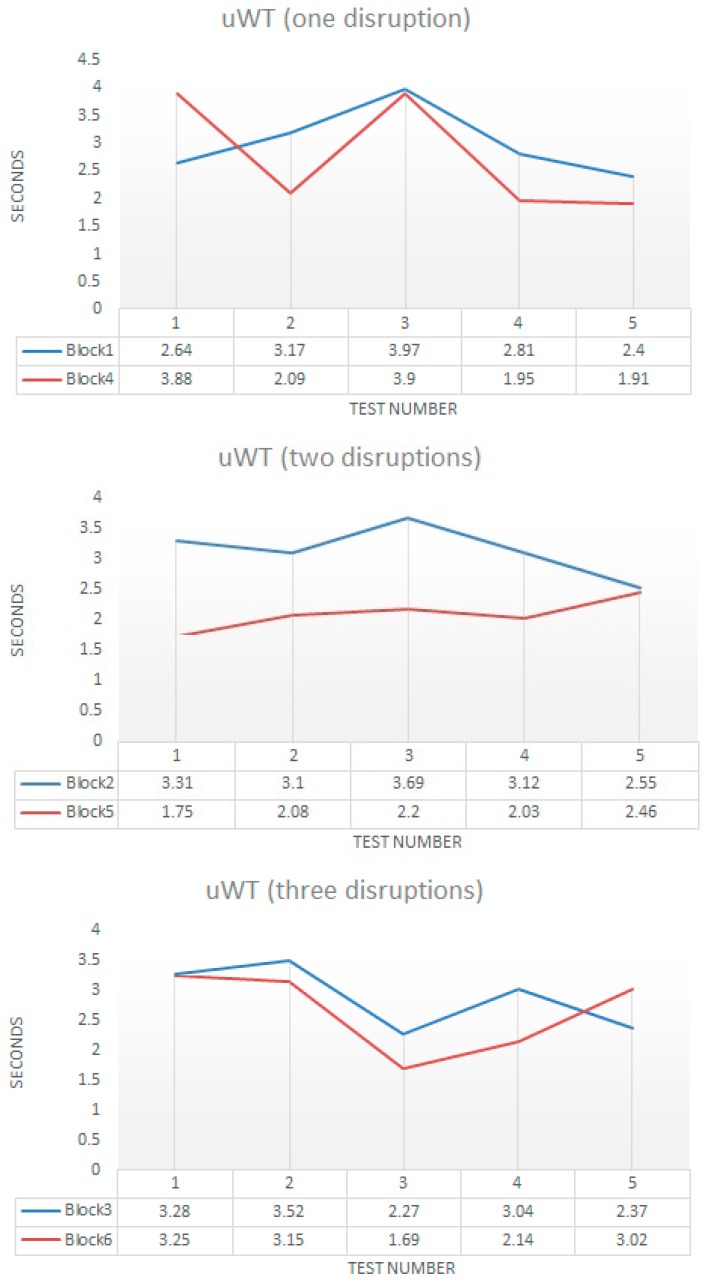
Values of *uWT* for one, two and three disruptions in the WTerm1 and WTerm2.

**Figure 15:**
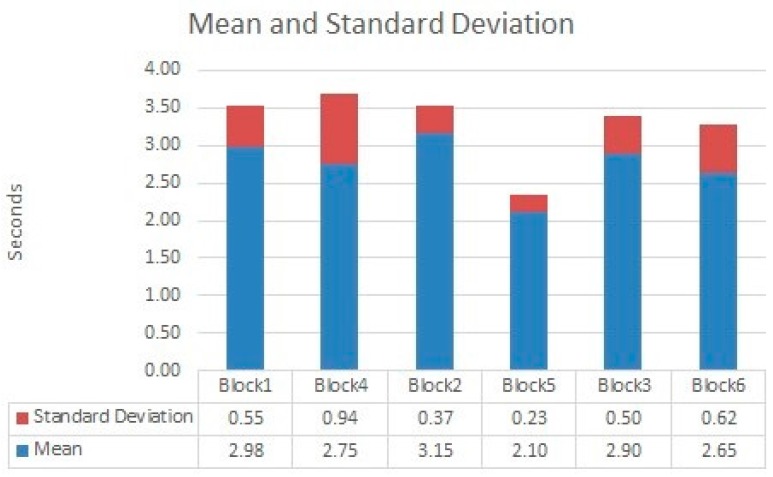
Mean and standard deviation for values of *uWT*.

Figure 14 shows the values for *uWT* obtained for all blocks and tested in each block. We grouped the blocks attending to the number of disruptions. For nearly all experiments, the *uWT* for WTerm 2 was less than for the WTerm 1 (only the test number two was greater in WTerm 2 than in WTerm 1). This was due to the differences of bandwidth of WiFi and power processing of both terminals. Figure 15 shows the mean and standard deviation for each block. Only for two disruptions, the mean value was significative. However, in the rest of disruptions the differences are minimal. This is important because it showed that our mitigation mechanism was independent of the kind of wireless terminal. The standard deviation was relatively small which indicates a small deviation of values from the mean. This was interesting because showed the regular behavior of the mitigation mechanism for the different experiments.

The best news for *uWT* were that their maximum values were nearly less than 3 s (only in one case was greater but close to 3 s). This means that our mechanism is relatively fast and it will allow the guard to receive video and reconnect after a disruption quickly.

**Figure 16:**
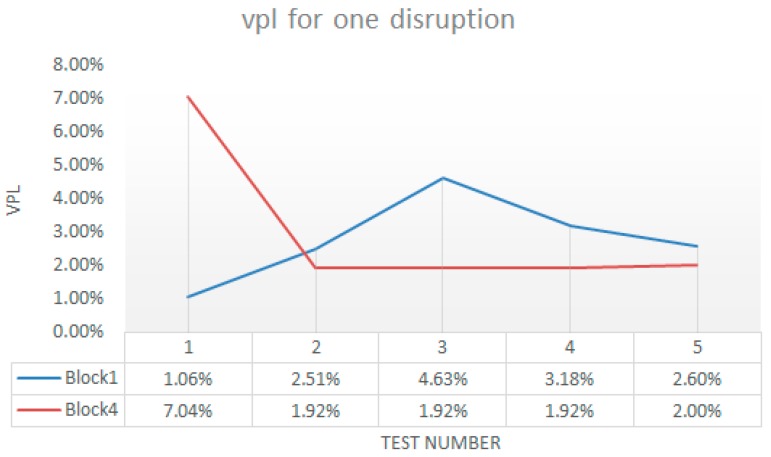

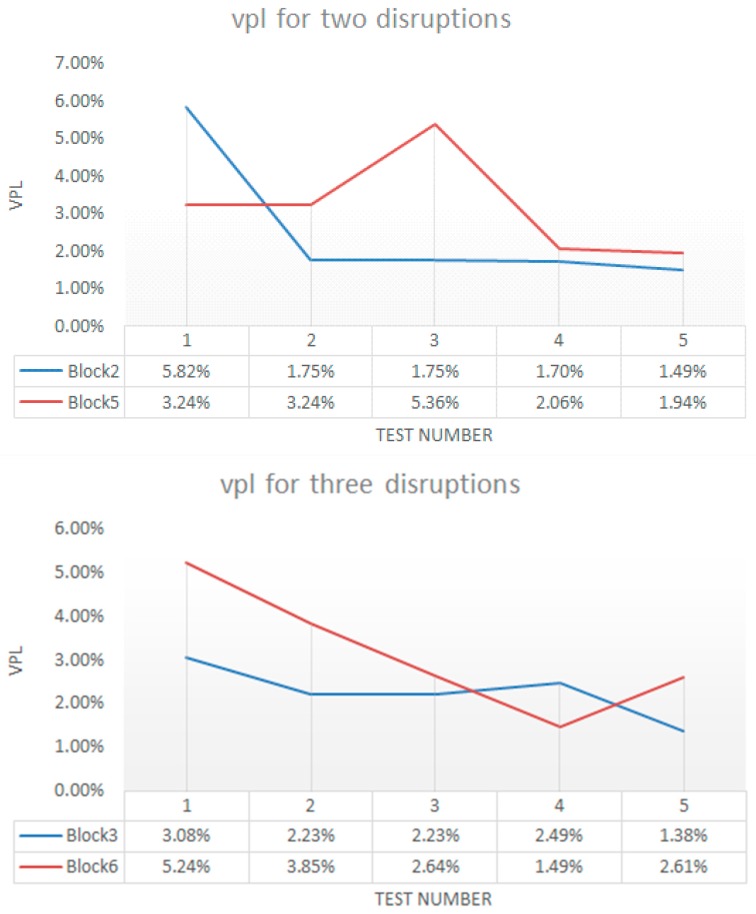
Values of *vpl* for one, two and three disruptions in the WTerm1 and WTerm2.

**Figure 17:**
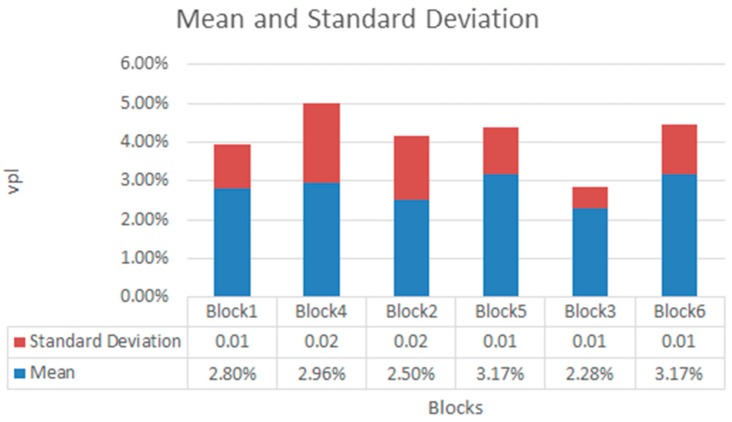
Mean and standard deviation for values of *vpl*.

Figure 16 shows the values for *vpl* obtained for all blocks and tested in each block grouped by number of disruptions. There was nor correlation between the kinds of wireless terminals neither number of disruptions and *vpl*. This was because the loss of packets depends on the WiFi channel, not on the terminal. Figure 17 shows the mean and standard deviation for each block. The standard deviation was very small which shows high regularity in the experiments.

The best news for *vpl* were that their maximum values were nearly less than 3%. This means that our mitigation mechanism worked notably. This value of *vpl* could justify that in all cases we obtained *WpR = 0*. One important tip was that when disruptions last little time (less than 10 s), it was confortable to continue with video visualization. However, when the total visualization time reached 400 s (disruptions last more time) it was not confortable to continue visualizing the video.

The above values of *uWT*, *vpl* and *WpR* are in consonance with the MOS.

### 5.3. Notification Messages, Handover and Battery Consumption

In Section 5.1, we showed all the messages of intrusion notification arrived to the user. In this Section, we show the numerical time of arrival of those messages to the user. We conducted 24 different tests measuring the time of arrival for e-mail and Telegram notification messages. We used the LTE and WiFi networks. We measured all the times using a chronometer of an Iphone Smart Phone. Those values were not exact values but were significative values because we mainly wanted to reproduce the user experience.

**Figure 18:**
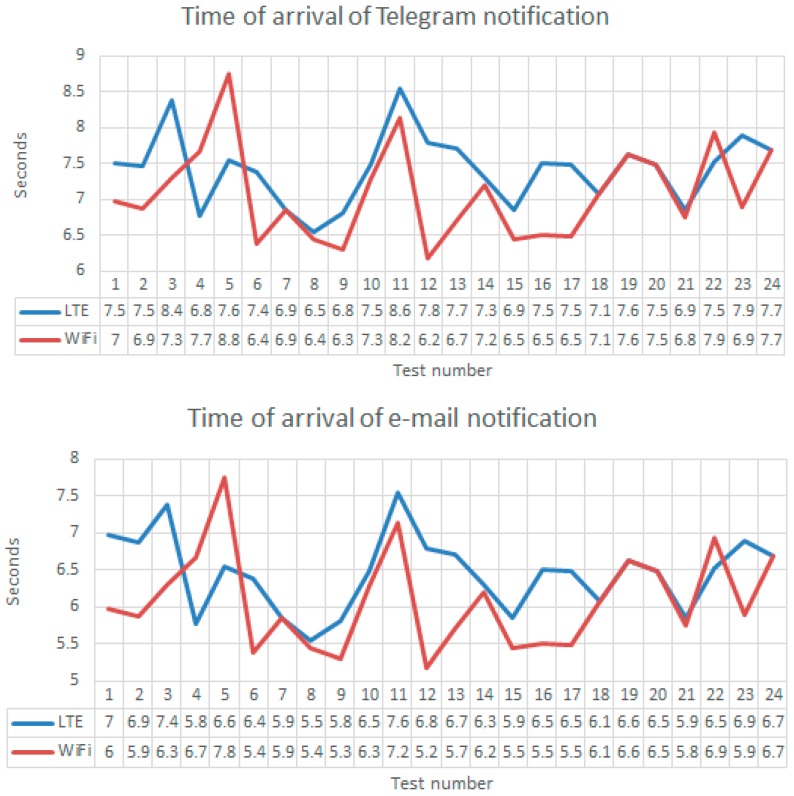
Time of arrival of notification messages of e-mail and Telegram using LTE and WiFi.

Figure 18 shows all those times of arrival. The mean value of notification message for Telegram (7.42 s for LTE and 7.08 s for WiFi) was around 1 s greater than e-mail notification message (6.46 s for LTE and 6.08 s for WiFi). The standard deviation was small: 0.48 for LTE and 0.64 for WiFi for Telegram notification message, and 0.5 for LTE and 0.64 for WiFi for e-mail notification messages. In WiFi, those standard deviations were greater than in LTE because WiFi networks provided a poorer QoS than LTE. The interesting news were that the time of arrival of notification messages are always less than 8 s. Therefore, in *uWT + 8 + G* (s) the guard will receive the first images of the intrusion, where *G* is the time needed by the guard to discover the notification message and to click the link. We consider that in less than 20 s the guard could receive the intrusion images. That is, the guard will experiment a delay of 20 s about the movements of the intruder.

**Figure 19:**
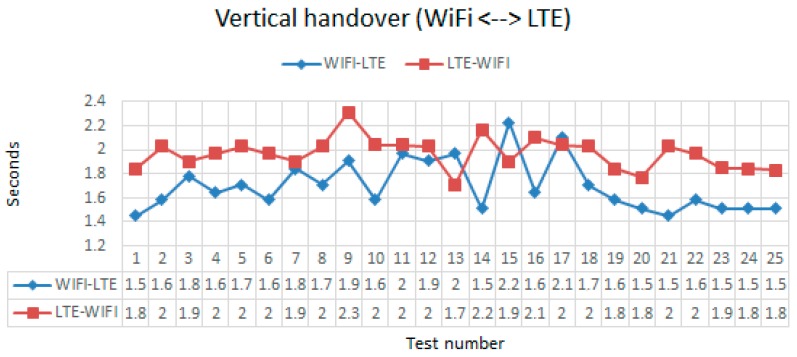
Time spent in the vertical handover (LTE←→WiFi).

Another source of delay in the reception of the first images of the instrusion and disruptions are the time needed to make the vertical handover between LTE and WiFi (or viceversa). We conducted other 24 tests to measure those delays with the Iphone Chronometer. Figure 19 shows the very small times of handover (perceived by the user because we measured them in observing the effect of the handover in the user Smart Phone). The mean of handover from WiFi to LTE is 1.6 s. That value was less than the 1.9 s needed to do the handover from LTE to WiFi. However, the standard deviation is greater in the first case (0.21) than in the second case (0.13). This was because WiFi Qos is pooerer than LTE QoS. The important news are that those times are very small. So the influence of handover was considered as a very small disruption that could be always mitigated.

It is very difficult to measure the influence of the redundant packets of video on the battery consumption. That is because the consumption of the battery does not follow a linear model, and not necessary all the redundant messages would consume the same amount of energy. We conducted several informal experiments in which annotated the amount of remaining hours of battery energy every 2 min for a video lasting 20 m. Then we introduce 3 disruptions and repeated the experiment. We found that in average the variation of the remaining amount of minutes was 2. That is, with the disruptions we lost 2 min of battery energy.

## 6. Conclusions

In this paper, we have shown the performance of a technique to mitigate the real time video streaming service disruptions applied to a video surveillance system in a Smart Home model. We have proposed novel ideas and implementations of those ideas. The intelligent protocol communication among sensors, Alarm processor, video server and the client allow our system to mitigate disruptions. The novelty that represents to demonstrate that it is possible to deliver real time video of the intrusion to the moving guard is important. We have shown that our system is fast, it has very low cost and is programmed and implemented using a systematic hardware and software design (based on standard software design patterns) and implementation. The most important result is that the satisfaction of the interviewees was highly shown. Another important result is that informally we have shown that it is possible to save energy consumption in the Smart Phone.

Future work includes more research in energy saving in the Smart Phone. We plan to design a formal model of energy saving for our system. We will consider battery drop models, formalization of energy wasted in sending a video packet and receiving it in the Smart Phone and the design and implementation of specific operation for an Observer and Strategy patterns that could monitorize the energy consumtion in the Smart Phone. We will consider professional people of videovigilance systems in order to better validate our system.

## Figures and Tables

**Figure 9:**
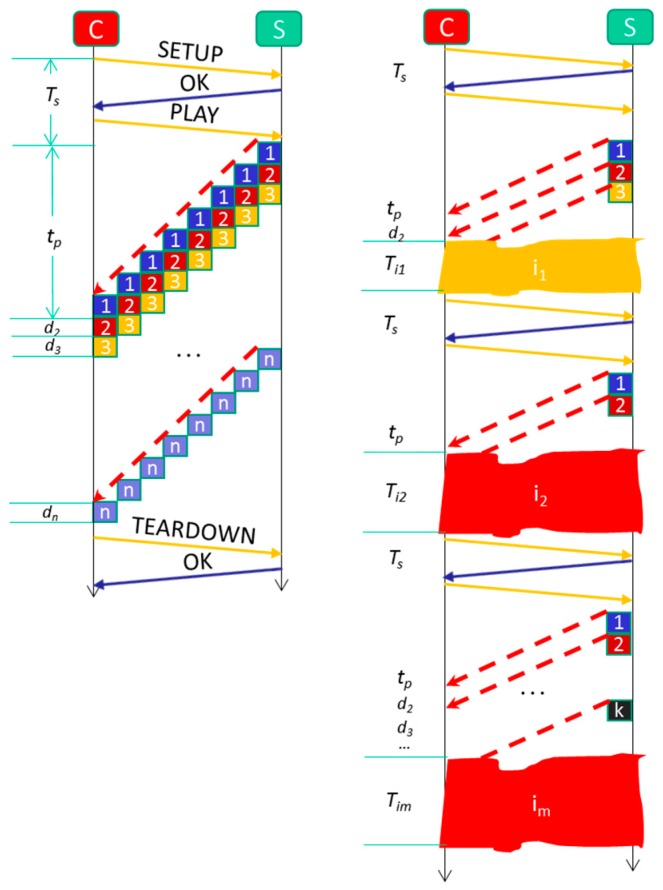
Communication times without and with video service disruptions.

**Figure 10:**
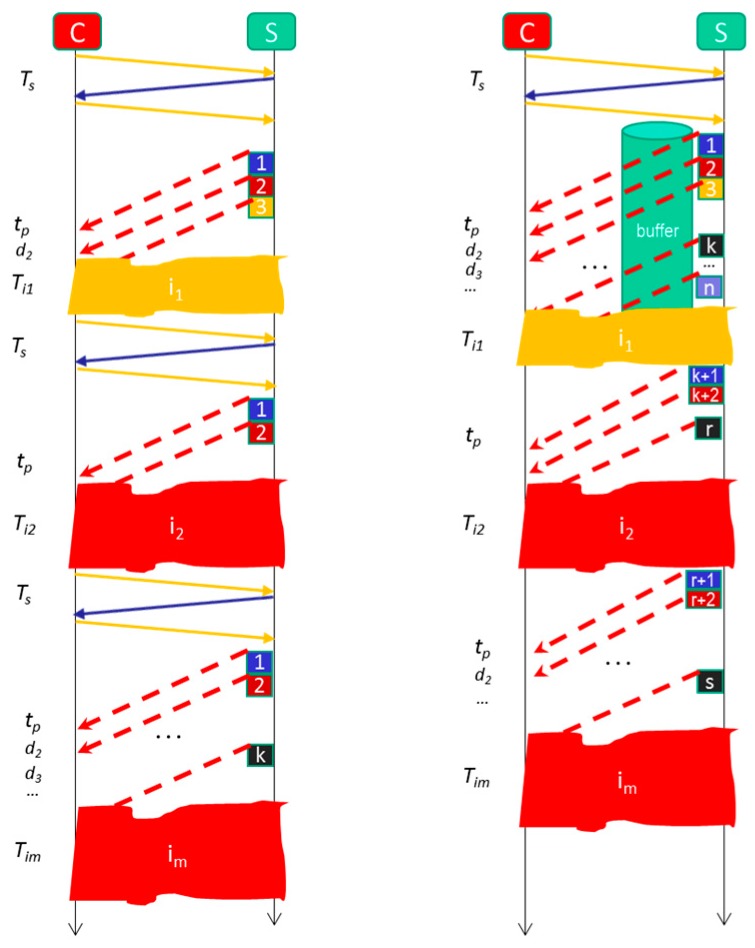
Communication times with video service disruptions and with the new method of mitigating the adverse effects of video service disruptions.

**Table 1:** Description of actors and use cases of Figure 2.

Actors	Alert Intrusion	Person that Invades the Home and Provokes an Intrusion
Use Cases	User	Guard in charge of process alert messages and visualize real time intrusion video
	Start Sensor Provider	Initiates the processing of the alarm after the sensors, asynchronously, have captured the information inside the Smart Home. Tests the sensors. Evaluates the range values. Triggers alarm.
	Start Message Procesor	Initiates the alert message processing, which constructs the messages to be sent to the *User*.
	Activate Telegram Bot Agent	Send the Telegram instant message to the *User*.
	Activate Mail Agent	Sends an e-mail to the *User*.
	Start Video Server	Initiates the Video Server to start the communication when requested by the *User*.
	Activate Recording Agent	Activates the agent that records the offline video.
	Activate Transmission Agent	Activates the agent that communicates the video to the *User*. Request the activation of the camera.
	Start video service	Starts the video session from the *User*. Visualize the video.

**Table 2:** Description of actors and use cases of Figure 4.

Actors	Video Interruption	Represents the Disruption Causes.
Use Cases	Recording Agent	Manages video recording during disruptions.
	Client	User or Entity that request to visualize the video.
	Transmission Agent	Manages video transmission and monitors the channel.
	Video Service	Video deployment to the User or Client.
	Test Transmission Channel	Evaluates the wireless channel state. When a video service disruption occurs an alarm is triggered, it will store the last not received video frames.
	Interrupt Video Service	Tests if a video Service interruption occured.
	Stop Transmission	Stops the video streaming service to the Client.
	Start Recording Offline Video	Once a video service interruption was detected, the video storing will start.
	Restart Transmision	Once the service interruption finalizes, the previous stored video will start to be sent to the Client.
	Stop Recording Offline Video	When video service interruption finalizes offline video will stop to be stored.
	Send Offline Video	Sends the video stored offline.
